# *PIK3CA* Mutations Frequently Coexist with *EGFR/KRAS* Mutations in Non-Small Cell Lung Cancer and Suggest Poor Prognosis in *EGFR/KRAS* Wildtype Subgroup

**DOI:** 10.1371/journal.pone.0088291

**Published:** 2014-02-12

**Authors:** Lei Wang, Haichuan Hu, Yunjian Pan, Rui Wang, Yuan Li, Lei Shen, Yongfu Yu, Hang Li, Deng Cai, Yihua Sun, Haiquan Chen

**Affiliations:** 1 Department of Thoracic Surgery, Shanghai Medical College, Fudan University Shanghai Cancer Center, Shanghai, China; 2 Department of Oncology, Shanghai Medical College, Fudan University Shanghai Cancer Center, Shanghai, China; 3 Department of Pathology, Fudan University Shanghai Cancer Center, Shanghai, China; 4 Department of Biostatistics, School of Public Health, Fudan University, Shanghai, China; UNIVERSITY MAGNA GRAECIA, Italy

## Abstract

**Purpose:**

*PIK3CA* gene encoding a catalytic subunit of the phosphatidylinositol-3-kinase (PI3K) is mutated and/or amplified in various neoplasia, including lung cancer. Here we investigated *PIK3CA* gene alterations, the expression of core components of PI3K pathway, and evaluated their clinical importance in non-small cell lung cancer (NSCLC).

**Materials and methods:**

Oncogenic mutations/rearrangements in *PIK3CA*, *EGFR*, *KRAS*, *HER2*, *BRAF, AKT1* and *ALK* genes were detected in tumors from 1117 patients with NSCLC. *PIK3CA* gene copy number was examined by fluorescent *in situ* hybridization and the expression of PI3K p110 subunit alpha (PI3K p110α), p-Akt, mTOR, PTEN was determined by immunohistochemistry in *PIK3CA* mutant cases and 108 patients without *PIK3CA* mutation.

**Results:**

*PIK3CA* mutation was found in 3.9% of squamous cell carcinoma and 2.7% of adenocarcinoma. Among 34 *PIK3CA* mutant cases, 17 tumors harbored concurrent *EGFR* mutations and 4 had *KRAS* mutations. *PIK3CA* mutation was significantly associated with high expression of PI3K p110α (*p*<0.0001), p-Akt (*p* = 0.024) and mTOR (*p* = 0.001), but not correlated with *PIK3CA* amplification (*p* = 0.463). Patients with single *PIK3CA* mutation had shorter overall survival than those with *PIK3CA*-*EGFR/KRAS* co-mutation or wildtype *PIK3CA* (*p* = 0.004). A significantly worse survival was also found in patients with *PIK3CA* mutations than those without *PIK3CA* mutations in the *EGFR/KRAS* wildtype subgroup (*p* = 0.043)

**Conclusions:**

*PIK3CA* mutations frequently coexist with *EGFR/KRAS* mutations. The poor prognosis of patients with single *PIK3CA* mutation in NSCLC and the prognostic value of *PIK3CA* mutation in *EGFR/KRAS* wildtype subgroup suggest the distinct mutation status of *PIK3CA* gene should be determined for individual therapeutic strategies in NSCLC.

## Introduction

It has been well established that the phosphatidylinositol-3-kinase (PI3K) pathway is related to carcinogenesis in a variety of human cancers [1], [2], [3]. Upon activation, PI3K initiates events leading to phosphorylation of Akt, which affects additional downstream signaling proteins involved in cell growth, metabolism, proliferation, survival, motility, and invasion [4], [5], [6]. PI3K-dependent activity is frequently elevated due to mutation of *PIK3CA*, a gene encoding the p110α catalytic subunit of PI3K (PI3K p110α), and the absence of the phosphatase and tensin homolog(PTEN) protein, a tumor suppressor with an important role in regulating the PI3K antiapoptotic and survival pathway [7], [8]. In addition, increased copy number of *PIK3CA* is also shown to be associated with increased *PIK3CA* transcription, p110α protein expression and PI3-kinase activity [9].

Aberrations in the components of the PI3K signaling pathway have been reported in many solid tumors, including lung cancer [2], [4], [7], [9]. Multiple mutations of *PIK3CA* that occur with regularity and in highly conserved regions of the gene lead to amino acid substitutions in the helical binding domain encoded by exon 9 and in the catalytic subunit of p110α encoded by exon 20, which result in upregulating PI3K pathway signaling [10]. In lung cancer, copy number gains of *PIK3CA* were found to be exclusive to *PIK3CA* mutation, implying that both alterations may have oncogenic potential to promote carcinogenesis in the lung [11]. The PTEN protein negatively regulates the PI3K pathway [12] and loss of PTEN protein expression has been linked to poor survival in patients with tongue cancer, and with more advanced tumor in esophageal and oral squamous cell cancers, respectively [13], [14]. Furthermore, Akt and mTOR lie downstream of PI3K and increased mTOR phosphorylation is frequently observed alongside with activated Akt in NSCLC and dysregulation of mTOR contributes to lung cancer progression [15], [16].

In our previous study, we reported that 90% of 52 lung adenocarcinoma samples from East Asian never smokers harbored driver mutations in just *EGFR*, *KRAS*, *HER2* and *ALK* genes [17]. The frequency of *EGFR*, *KRAS*, *HER2* mutations and *EML4-ALK* fusion were 75.3%, 2%, 5.9% and 5%, separately, in recent analysis of 202 lung adenocarcinoma samples from Chinese patients who never smoked [18]. However, no more than 40% of cases of NSCLC which includes squamous cell carcinoma, adenocarcinoma and large cell carcinoma histology would harbor such alterations [18]. It is now evident that even within a clearly identifiable histologic subtype, distinct molecular changes may be associated with a spectrum of clinical characteristics and also correlate with disease outcome and response to treatment [19]. As a result, it will be necessary to clarify different molecular alteration for individual treatment.

To date, there are few studies that constitute a comprehensive picture of the expression of components in PI3K pathway, *PIK3CA* gene alteration, and their correlation to NSCLC [20], [21]. In the present study, we examined *PIK3CA* gene mutation, *PIK3CA* amplification as well as the expression of PI3K p110α, p-Akt, mTOR and PTEN which lie in the PI3K pathway in a consecutive collection of NSCLC tumor samples. This detailed understanding of PI3K pathway alterations in NSCLC might enable a more precise delineation of candidate target populations, facilitating clinical trial design and validation of predictive biomarkers.

## Materials and Methods

### Patients and samples

From October 2007 to December 2012, we consecutively procured primary tumor samples from NSCLC patients who underwent pulmonary resection at the Department of Thoracic Surgery, Fudan University Shanghai Cancer Centre. Subjects eligible for this study had to meet the following: pathologically confirmed lung adenocarcinoma or lung squamous cell carcinoma, each sample containing sufficient tissue for comprehensive mutational analyses and with no neoadjuvant treatment. Patients were followed up every 3 months for the first 2 years, then biannually thereafter. A contrast-enhanced chest computed tomography (CT) scan was taken every 3 months for the first 2 years and then every 6 months thereafter. If recurrence was suspected either through newly presenting symptoms or through scheduled tests, integrated positron emission tomography/CT (PET/CT) was performed. PET/CT was also taken in patients without symptoms or abnormal findings in the scheduled tests 1 year after the surgical resection. Final diagnosis of recurrence was confirmed by the histopathological examination of samples obtained from surgery or biopsy. If it was impossible to diagnose the recurrence histopathologically, recurrent malignancy was no longer suspected based on the clinical and radiological follow-up period of at least 12 months with no evidence of active malignancy. This research was approved by the Institutional Review Board of the Fudan University Shanghai Cancer Center. Written informed consent was obtained from all patients.

### Mutational analyses

Frozen tissue of tumor specimens was grossly dissected into TRIZOL (Invitrogen, Life Technologies, Carlsbad, CA), followed by total RNA extraction using standard protocol. Total RNA samples were reverse transcribed into cDNA. *PIK3CA* exon 9 includes codons 542 and 545 and *PIK3CA* exon 20 includes codon 1047, where the majority of mutations occur [22]. So we searched for mutations in *PIK3CA* exons 9 and 20. *EGFR* (exons 18–21), *HER2* (exons 18–21), *KRAS* (exons 2–3), *BRAF* (exons 11–15) and *AKT1* (exons 2–3) were also amplified by PCR using cDNA. Amplified products were analyzed by direct dideoxynucleotide sequencing. To identify *EML4*-*ALK* fusions, multiple 50 primers were used along with a fixed 30 primer localizing to *ALK* exon 20 to detect all known *EML4* fusion variants as previously described [17]. Primers used to detect fusions between *ALK* and *KIF5b* or *TFG* were as previously reported [23], [24]. *ALK* FISH was also used to confirm the accuracy of PCR. All mutated cases were confirmed twice with independent PCR reactions. New data was not generated in our study.

### Expression of core components of PI3K pathway

Tumoral tissue selection and immunohistochemial evaluation were performed by two pathologists (Yuan L and Lei S). Surgical specimens were fixed in 10% formalin and embedded in paraffin; 4-µm sections from lung tumors were prepared and deparaffinized, and antigen retrieval was done by microwaving. Endogenous peroxidase activity was blocked with 0.3% H_2_O_2_. After blocking with normal serum, sections were incubated for 120 minutes with monoclonal antibodies against PI3K p110α (1∶400 dilution, clone: C73F8; Cell Signaling Technology), PTEN (1∶50 dilution, clone: 138G6; Cell Signaling Technology), p-Akt (1∶50 dilution, clone: Ser473; Cell Signaling Technology) and mTOR (1∶50 dilution, clone: 7C10; Cell Signaling Technology). Slides were washed in PBS and detected with horseradish peroxidase conjugated anti-rabbit/mouse Real Envision Detection kit (Gene Tech), followed by counterstaining with hematoxylin.

According to scoring system that has been reported previously in the literature [25], [26], Our PI3K p110α staining scoring was done as follows: The score was 0 if no positive tumor cells were found; 1 if positive tumor cells were <10%; and 2 if positive tumor cells were >10%. Tissues with scores of 0 or 1 were considered low expression; those with scores of 2 were considered high expression. Immunoreactivity of p-Akt, mTOR and PTEN was evaluated semiquantitatively based on staining intensity and proportion, as previously described [27]. Staining intensity was scored as: absent (0); weak (1); moderate (2), or strong staining (3). Staining proportion was scored as: none (0); less than 1/3 (1); 1/3 to 2/3 (2), or more than 2/3 of tumor cells (3). The overall score was calculated as the sum of the intensity score and the proportion score, yielding a score between 0 and 6. An overall score of 0–2 was regarded as low expression while the other scores were regarded as high expression statistical analysis. Immunohistochemical staining was independently evaluated by two pathologists (Yuan L and Lei S). In cases of different overall scores taken from the same tumor, the average score was considered the final overall score.

### Assessment of *PIK3CA* gene amplification

Fluorescent in situ hybridization (FISH) assay for *PIK3CA* was performed by using *PIK3CA* probe that hybridizes to the band 3q26.32 with Texas Red (red) and centromere3(CEN3) with FITC(green) (Abbott Molecular, Abbott Park, IL) following routine methods. FISH analyses was interpreted by two experienced evaluators (Lei W and Yunjian P) blinded to the clinical data. At least 100 nuclei per patient were evaluated. Tissue samples with a *PIK3CA*/CEN3 ratio of 1.0 were classified as normal and those with a *PIK3CA*/CEN3 ratio between 1.0 and 2.0 were classified as having *PIK3CA* gains. A *PIK3CA*/CEN3 ratio of more than 2.0 was considered amplified. A minimum of 50 cells with both centromeric and *PIK3CA* gene signals were scored to give conclusive data.

### Statistical analysis

Difference in proportions was analyzed by *X^2^* or Fisher's exact test. Recurrence-free survival (RFS) duration was defined as the time of surgery to recurrence or last contact. Overall survival (OS) was defined as the time of surgery to death or last contact. Event was stated as recurrence or death since surgery. Patients alive and showing no recurrence at the last follow-up were censored. RFS and OS distributions were estimated using the Kaplan-Meier method. The log-rank test was used to determine survival differences between groups. Regression analyses of survival data, based on the Cox proportional hazards model, were conducted on RFS and OS. A forward stepwise selection procedure was implemented with a *P*-value threshold of 0.05 for inclusion in the multivariate analysis. Statistical significance was accepted when the *P*-value was <0.05. All data were analyzed using the Statistical Package for the Social Sciences Version 16.0 Software (SPSS Inc., Chicago, IL).

## Results

### *PIK3CA* gene mutation status in NSCLC

**Figure 1:**
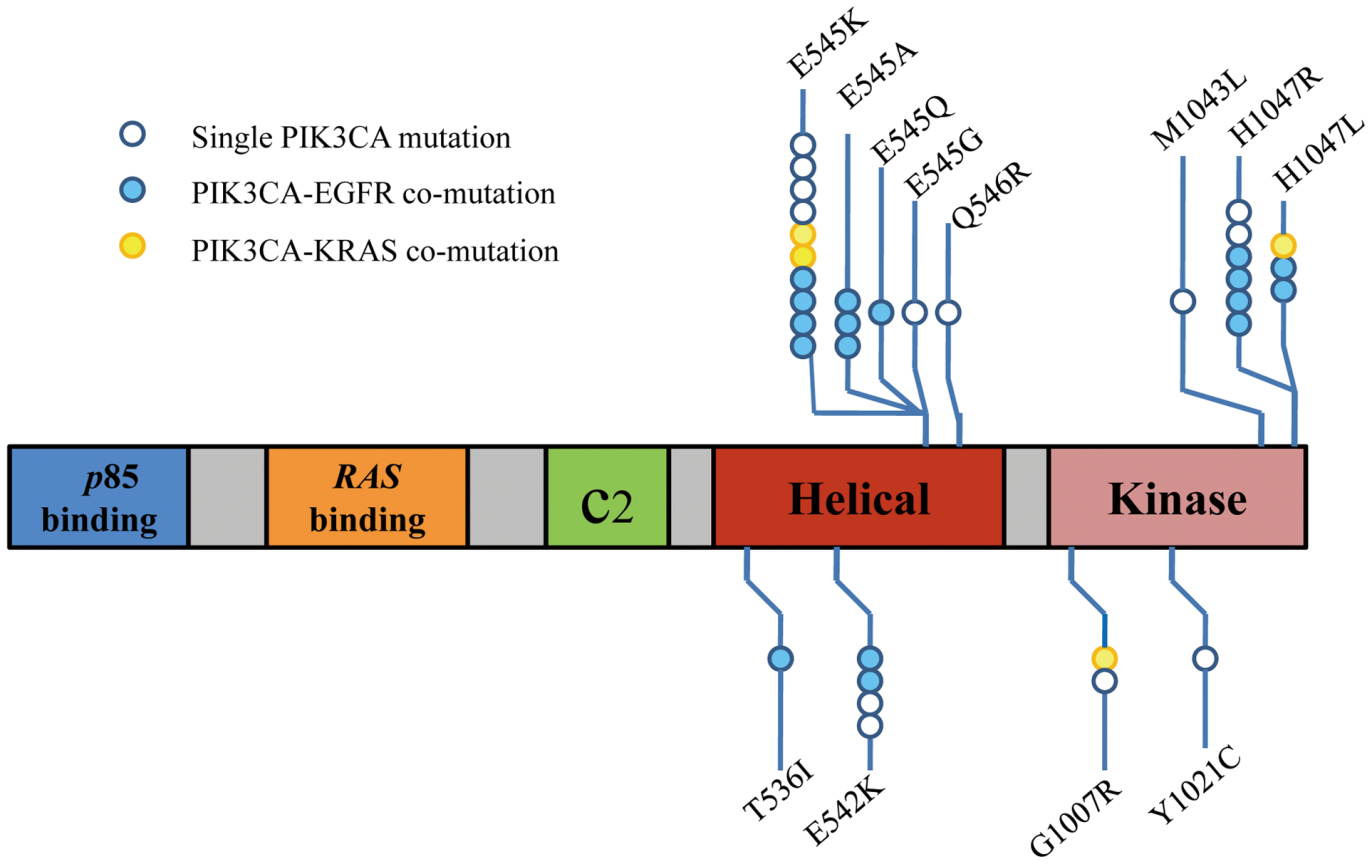
Mutation in *PIK3CA*. Boxes represent functional domains (the p85 binding domain, Ras binding domain, C2 domain, helical domain, and kinase domain). Frequency and different types of mutations detected within each region is indicated below and above the box.

A total of 1,117 NSCLC patients including 646 men and 471 women were eligible for mutation analysis in this study. As presented in **Table 1**, **Figure 1** and **Figure S1 in File S1**. 3.0% (34/1117) patients harbored mutations in *PIK3CA*, accounting for 2.7% (22/807) of lung adenocarcinomas, and 3.9% (12/310) of squamous cell carcinomas. No significant correlation was observed between *PIK3CA* mutations and clinicopathological factors such as gender, age, pathological types, smoking history, tumor differentiation or stage (**Table 1**).

**Table 1:** Clinicopathological data of 1117 NSCLC patients.

Factors	Mutations patients Positive(%)	Wild type patients Positive(%)	*p*-Value
Stage			
I	13(38.2)	544(50.2)	0.168
II∼IV	21(61.8)	539(49.8)	
Lymph node metastasis			
N0	15(44.1)	613(56.6)	0.149
N+	19(55.9)	470(43.4)	
Smoking history			
Never smoker	16(47.1)	582(53.7)	0.442
Current or former smoker	18(52.9)	501(46.3)	
Differentiation			
Well	4(11.8)	150(13.9)	0.728
Moderately or poorly	30(88.2)	933(86.1)	
Pathological types			
AD	22(64.7)	785(72.4)	0.319
SCC	12(35.3)	298(27.5)	
Age			
≤60	21(61.8)	599(55.3)	0.456
>60	13(38.2)	484(44.7)	
Gender			
Male	21(61.8)	625(57.7)	0.637
Female	13(38.2)	458(42.3)	
Chemotherapy*			
Adjuvant chemotherapy	20(58.8)	62(57.4)	
No chemotherapy	14(41.2)	46(42.6)	0.884

N+: lymph node metastasis positive; AD: adenocarcinoma; SCC: squamous cell carcinoma;

* Chemotherapeutic analysis based on 34 *PIK3CA* mutant patients and 108 *PIK3CA* wildtype patients.

Mutations in exon 9 encoding for the helical domain (E545K, E545Q, E545G, E545A, Q546R, E542K, T536I) were found in 21 patients. Exon 20 mutations coding for the kinase domain (H1047R, H1047L, M1043L, G1007R, Y1021C) were found in 13 patients. The most frequent mutations were E545K and H1047R occurring in 16(47.1%, 16/34) of 34 patients with *PIK3CA* mutations (**Figure 1**, **Table 2**). According to clinicopathologic data identified, analysis of frequency of mutations in the helical vs. kinase domain was carried out. There was a trend that more helical domain *PIK3CA* mutations were observed in patients with lymph node metastasis staged II∼III, but the difference did not reach statistical significance (**Table S1 in File S1**).

**Table 2:** Clinicopathological data about 34 *PIK3CA* mutant patients.

*PIK3CA*	Other	Age	Sex	Tobacco use	pN	pStage	Pathology	Differentiation	*PIK3CA* amplifivation
E545K	EGFR	50	F	N	N0	Ia	AD	Moderate	Not amp
E545Q	EGFR	60	M	C/F	N1	IIIa	AD	Poor	Not amp
E545A	EGFR	40	M	N	N0	Ia	SCC	Moderate	Not amp
E545A	EGFR	57	F	N	N0	Ia	AD	Moderate	Not amp
E542K	EGFR	65	M	C/F	N2	IIIa	AD	Moderate	Not amp
E545K	EGFR	63	M	C/F	N0	IIa	AD	Moderate	Not amp
E545A	EGFR	56	M	C/F	N2	III a	AD	Moderate	Not amp
T536I	EGFR	64	F	N	N1	IIb	AD	Well	Not amp
E545K	EGFR	63	F	N	N2	IIIa	AD	Moderate	Not amp
E545K	EGFR	65	F	N	N2	IIIa	AD	Poor	Not amp
E542K	EGFR	54	F	N	N2	IIIa	AD	Moderate	Not amp
H1047L	EGFR	41	F	N	N2	IIIa	AD	Moderate	Not amp
H1047R	EGFR	55	M	N	N2	IIIa	AD	Moderate	Not amp
H1047R	EGFR	58	F	N	N0	Ia	AD	Moderate	Not amp
H1047R	EGFR	34	F	N	N0	Ia	AD	Poor	Not amp
H1047R	EGFR	52	M	C/F	N1	Ia	AD	Moderate	Not amp
H1047L	EGFR	65	F	N	N0	Ia	AD	Well	Not amp
E545K	KRAS	58	M	C/F	N0	Ia	SCC	Moderate	Not amp
E545K	KRAS	58	M	C/F	N2	IIIa	AD	Moderate	Not amp
H1047L	KRAS	49	F	N	N0	Ib	AD	Moderate	Not amp
G1007R	KRAS	59	M	C/F	N2	IIIa	AD	Poor	Not amp
E545K		58	M	C/F	N0	Ia	SCC	Moderate	Not amp
Q546R		57	M	C/F	N2	IIIa	AD	Poor	Not amp
E542K		54	M	C/F	N2	IIIa	SCC	Moderate	Amp
E545G		76	M	N	N2	IIIa	SCC	Poor	Amp
E545K		75	M	C/F	N0	Ia	SCC	Moderate	Not amp
E545K		68	M	C/F	N0	IIa	SCC	Well	Amp
E542K		68	M	C/F	N2	IIIa	AD	Moderate	Not amp
E545K		72	M	N	N1	IIb	SCC	Moderate	Not amp
Y1021C		72	M	C/F	N0	Ia	SCC	Moderate	Amp
M1043L		60	M	C/F	N0	IIa	SCC	Moderate	Not amp
H1047R		59	M	C/F	N1	IIa	SCC	Well	Not amp
G1007R		75	F	C/F	N2	IIIa	SCC	Moderate	Not amp
H1047R		38	F	N	N0	Ia	AD	Moderate	Amp

F:female; M:male; N:never smoker; C/F:current or former smoker; AD:adenocarcinoma; SCC:squamous cell carcinoma; Amp:amplification.

### *PIK3CA* mutations frequently coexist with *EGFR/KRAS* mutations in NSCLC

To explore the coexistence of *PIK3CA* and other oncogene mutations in non-small cell lung cancer, testing for *EGFR*, *KRAS*, *HER2*, *BRAF*, *AKT1* gene mutations and *ALK* rearrangement was also arranged. *EGFR* and *KRAS* gene mutations were found in 536 (48.0%, 536/1117) and 67(6.0%, 67/1117) of 1117 patients. The occurrence rates of *HER2*, *BRAF*, *AKT1* mutations and *ALK* rearrangement were 20(1.8%, 20/1117), 11(1%, 11/1117), 2(0.2%, 2/1117) and 33(3.0%, 33/1117) respectively. All of the identified *ALK* fusion variants were *EML4-ALK*. Other fusion variants such as *KIF5B-ALK* and *TFG-ALK* were not found in our study. Complete data about *ALK* rearrangement was shown in **Table S2 in File S1**. No correlation was found between *PIK3CA* mutations and other gene alterations neither in lung squamous cell carcinoma nor in adenocarcinoma groups (**Tabl**e **S3–S4 in File S1**).

**Figure 2:**
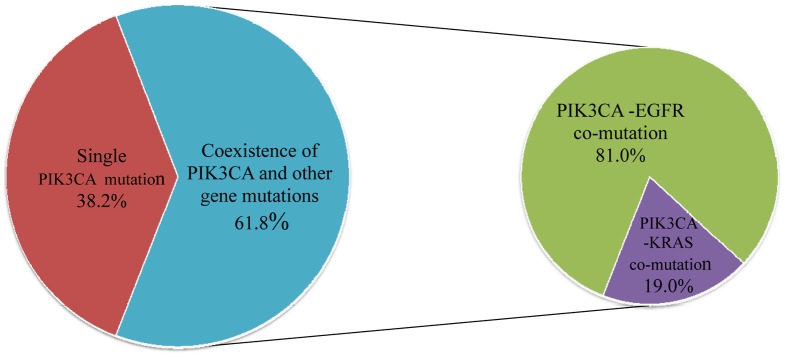
Coexisting mutations in patients with *PIK3CA*-mutant NSCLC.

Among 34 *PIK3CA* mutant cases, twenty one (61.8%, 21/34) harbored concurrent oncogenic mutations — 17(81.0%, 17/21) *EGFR* mutations and 4(19.0%, 4/21) *KRAS* mutations (**Figure 2**), accounting for 3.2% (17/536) of the *EGFR*-mutant cases and 6.0% (4/67) of the *KRAS*-mutant cases. Coexistence of *PIK3CA* with *BFAF*, *HER2*, *AKT1* gene mutations or *ALK* rearrangement was not found. This *PIK3CA*-*EGFR/KRAS* co-mutation was more common in never smokers than in current or former smokers (*p* = 0.039), and in adenocarcinoma than in squamous cell carcinoma (*p*<0.0001). There was also a tendency toward a higher co-mutation ratio in females (*p* = 0.067) and in patients aged not more than 60 (*p* = 0.168) (**Table S5 in File S1**).

Specific histopathological subtype was also analysed in 807 lung adenocarcinoma and no significant difference in subtype was found between patients with and without *PIK3CA* mutation (*p* = 0.082, **Table S6 in File S1**). In 34 *PIK3CA* mutant cases, there was no significant correlation in histopathological subtype between patients with *PIK3CA*-*EGFR/KRAS* mutations and those only with *PIK3CA* mutations, either. (*p* = 0.121, **Table S7 in File S1**).

### Immunohistochemical expression of PI3K p110α, p-AKT, mTOR, PTEN and their correlations

To evaluate the activity of PI3K/AKT pathway both in *PIK3CA* mutant and *PIK3CA* wildtype groups, we consecutively selected a smaller series of *PIK3CA* wildtype patients with primary NSCLC, surgically resected between July 2008 and June 2009 with similar clinical and pathological characteristics from 1117 patients examined above. 108 *PIK3CA* wildtype patients were collected. We determined PI3K p110α, p-Akt, mTOR and PTEN protein levels in 34 *PIK3CA* mutant cases and 108 *PIK3CA* wildtype cases by immunohistochemistry (IHC). Representative immunostaining for each protein was illustrated in **Figure S2 in File S1**. For *PIK3CA* mutant group, high cytoplasmic expression of PI3K p110α was detected in 27(79.4%, 27/34) tumors, whereas high expression of p-Akt (mostly in cytoplasmic) and mTOR (in cytoplasmic) was detected in 18(52.9%, 18/34) and 25(73.5%, 25/34) tumors, respectively. Low expression of PTEN (PTEN loss) in the cytoplasm and nucleus was seen in 8(23.5%, 8/34) tumors. For *PIK3CA* wild type group, high expression of PI3K p110α, p-Akt, mTOR were found in 42(38.9%, 42/108), 34(31.5%, 34/108) and 44(40.7%, 44/108) tumors respectively. Low expression of PTEN was seen in 30(27.8%, 30/108) tumors. As shown in **Table S8–S9 in File S1**, in either of the two groups, the association between each pair of PI3K p110α, p-Akt, mTOR proteins were statistically significant. However, no significant correlations were found between PTEN and other proteins. In *PIK3CA* mutant group, high PI3K p110α expression was associated with stage II to IV disease. (*p* = 0.043) (**Table S8 in File S1**) and in *PIK3CA* wild type group more high p-Akt expression was found in older patients (*p* = 0.037) (**Table S9 in File S1**). None of other clinicopathologic characteristics showed a significant relationship with the expression of PI3K p110α, p-Akt, mTOR or PTEN.

### Analysis of *PIK3CA* gene amplification

To examine the copy number alterations of *PIK3CA*, the same panel of 142 frozen NSCLC tumor samples was analyzed by fluorescence in site hybridization including 34 tumors with mutation in the *PIK3CA* and 108 cases without *PIK3CA* mutation. *PIK3CA* amplification was detected in 5 *PIK3CA* mutant samples (14.7%, 5/34) which was more prevalent in lung squamous cell carcinoma (4/12 for lung squamous cell carcinoma vs. 1/12 for lung adenocarcinomas, *p* = 0.042). Nevertheless, in *PIK3CA* wild type group, *PIK3CA* amplification was detected in 22(20.4%, 20/108) tumors and was significantly associated with male gender (22/90 vs. 0/18, *p* = 0.021), current/former smoker (22/78 vs. 0/30, *p* = 0.001) and squamous cell carcinoma pathological type (19/52 vs. 3/56, *p*<0.0001). (**Table S10 and Figure S2 in File S1**)

### Association among *PIK3CA* mutation, *PIK3CA* amplification and the expression of PI3K p110α, p-AKT, mTOR, PTEN

We further determined whether *PIK3CA* alterations were associated with the activity of PI3K pathway. We observed that *PIK3CA* mutation was significantly associated with high expression of PI3K p110α (*p*<0.0001), p-Akt (*p* = 0.024) and mTOR (*p* = 0.001) (**Table 3**). However, no correlations were found between *PIK3CA* mutation and PTEN expression (*p* = 0.626) (**Table 3**). In addition, *PIK3CA* amplification was not correlated with the expression of PI3K p110α, p-AKT, mTOR, PTEN neither in *PIK3CA* mutant nor wild type group (**Table S10 in File S1**). We also compared *PIK3CA* amplification with *PIK3CA* mutation status and found that there were five cases harboring *PIK3CA* amplification in combination with *PIK3CA* mutations. No evident relation was found between *PIK3CA* mutation and amplification (*p* = 0.463) (**Table 3**)

**Table 3:** Correlation among *PIK3CA* mutation, amplification and expression of PI3K p110α, p-Akt,m TOR and PTEN.

Expression	*PIK3CA* mutation		*P*
	Positive(%)	Negative(%)	
PI3K p110α (+)	27(79.4)	42(38.9)	**<0.0001**
(−)	7(20.6)	66(61.1)	
PTEN loss (+)	8(23.5)	30(27.8)	0.626
(−)	26(76.5)	78(72.2)	
p-Akt (+)	18(52.9)	34(31.5)	**0.024**
(−)	16(47.1)	74(68.5)	
mTOR (+)	25(73.5)	44(40.7)	**0.001**
(−)	9(26.5)	64(59.3)	
*PIK3CA* amplification (+)	5(14.7)	22(20.4)	0.463
(−)	29(85.3)	86(79.6)	

### Survival outcomes according to *PIK3CA* gene mutation and amplification status

We further detect the prognostic value of *PIK3CA* gene mutation and amplification in the same panel of 142 cases. To clarify whether the therapies influenced over survival (OS) and recurrence-free survival (RFS), we compared the OS and RFS between patients with and without adjuvant chemotherapy, finding no significant difference between two groups neither on OS nor RFS (**Figure S3A–S3B in File S1**). To identify whether patients with *PIK3CA/EGFR* co-mutations could benefit from *EGFR* tyrosine kinase inhibitor, we also investigated the adjuvant therapy of 34 *PIK3CA* mutant cases. Of these, 2 patients, with L858R and 746-deletion separately, were treated with gefitinib after operation. Partial response was obtained in both of the two patients

**Figure 3:**
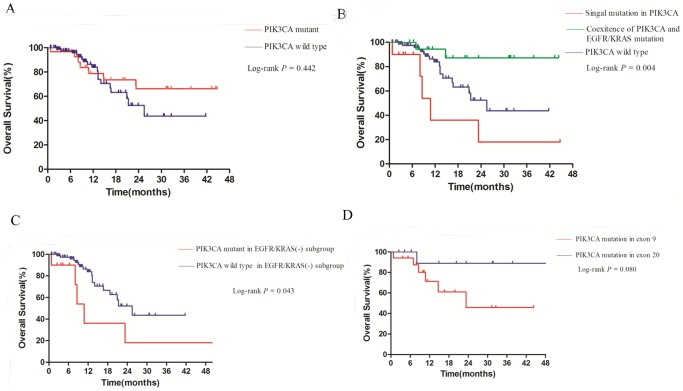
Overall survival curves for patients: with or without *PIK3CA* mutation (A); with single *PIK3CA* mutation, coexistence of *PIK3CA* and other gene mutation, and those in *PIK3CA* wild-type group (B); with or without *PIK3CA* mutation in *EGFR/KRAS* wild-type group (C); with *PIK3CA* mutation in exon 9 or exon 20(D).

The over survival (OS) for whole cohort was 12.0 months with a median follow-up time of 12.2 months. There was no significant difference in median OS between *PIK3CA* mutant group and *PIK3CA* wild type group (*p* = 0.442; **Figure 3A**). However, when *PIK3CA* mutant patients were sub-classified by single *PIK3CA* mutation or *PIK3CA*-*EGFR/KRAS* co-mutation, survival for patients with single *PIK3CA* mutation was shorter than patients with *PIK3CA*-*EGFR/KRAS* co-mutation or *PIK3CA* wild type group (*p* = 0.004; **Figure 3B**).

It has been well characterized that *EGFR* mutation was usually associated with a relatively good prognosis while *KRAS* mutation was shown to be associated with a poor outcome, leaving an indeterminate prognostic subgroup for the *EGFR/KRAS* wildtype patients. Therefore, we further investigated the prognostic role of *PIK3CA* mutations in *EGFR/KRAS* wildtype NSCLC patients. We found a significantly worse survival in patients with *PIK3CA* mutations (*p* = 0.043; **Figure 3C**). Besides, after *PIK3CA* mutant patients were allocated to E9 (*PIK3CA* mutation in exon 9) and E20 (*PIK3CA* mutation in exon 20) group, a trend was found that patient in E20 group survived longer than those in E9 group (*p* = 0.080; **Figure 3D**).

**Figure 4:**
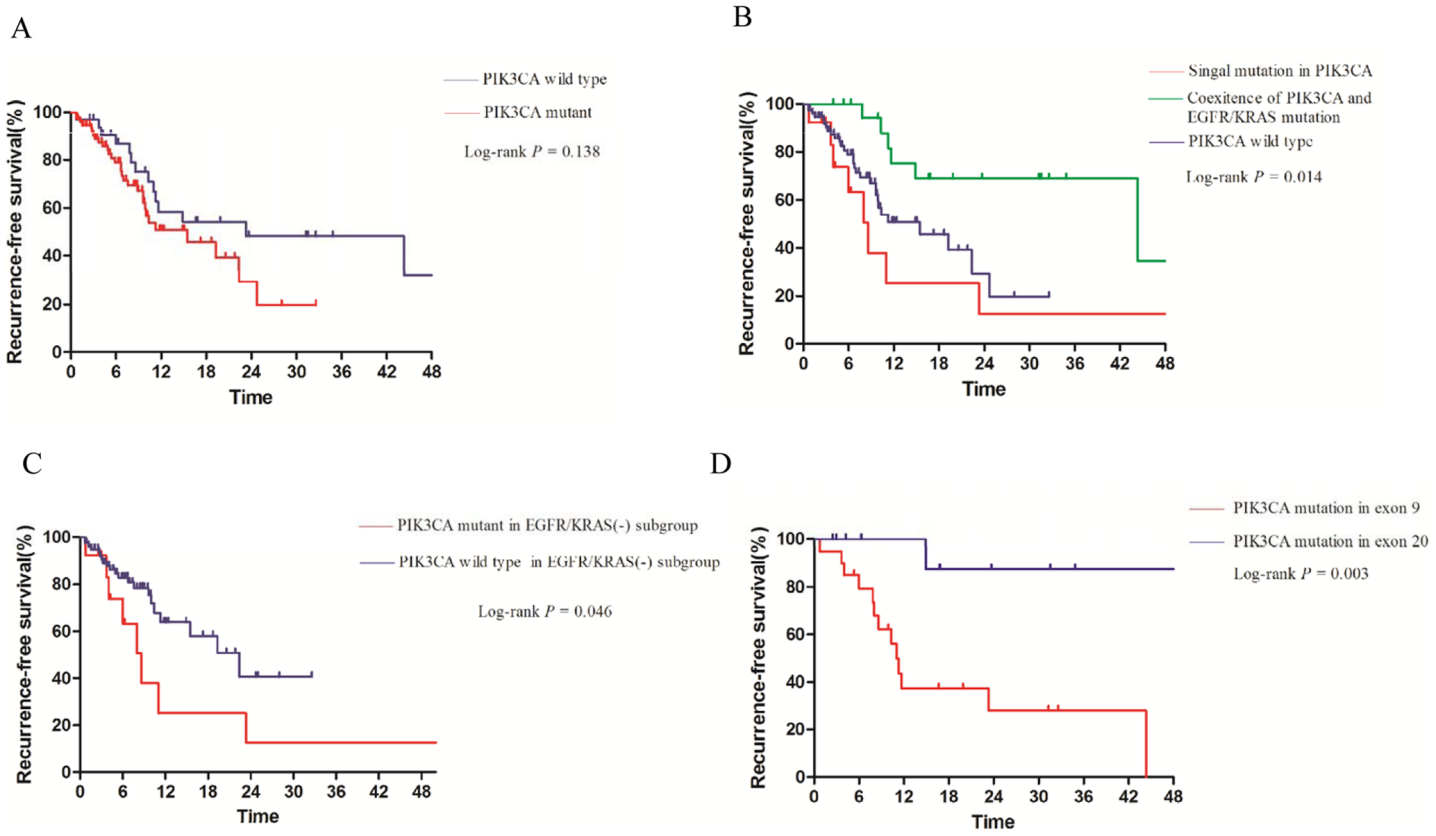
Recurrence-free survival curves for patients: with or without *PIK3CA* mutation (A); with single *PIK3CA* mutation, coexistence of *PIK3CA* and other gene mutation, and those in *PIK3CA* wild-type group (B). with or without *PIK3CA* mutation in *EGFR/KRAS* wild-type group (C); with *PIK3CA* mutation in exon 9 or exon 20 (D).

The median recurrence-free survival (RFS) was 15.5 months in patients with *PIK3CA* mutation and 23.3 months in patients without *PIK3CA* mutations (*p* = 0.138, **Figure 4A**). Like median OS, patients with single *PIK3CA* mutation had a shorter RFS than patients with *PIK3CA*-*EGFR/KRAS* co-mutation (*p* = 0.014, **Figure 4B**). Significant difference on RFS was also found between cases with and without *PIK3CA* mutation in *EGFR/KRAS* wildtype subgroup (*p* = 0.046; **Figure 4C**). Patients in E20 group had a longer RFS than those in E9 group (*p* = 0.003; **Figure 4D**). *PIK3CA* amplification was not associated with overall survival (*p* = 0.491; **Figure S4A in File S1**) or recurrence-free survival (*p* = 0.884; **Figure S4B in File S1**). We further performed a multivariate analysis (Cox proportional hazards) with *PIK3CA* mutation status, histological subtype, age, gender, tumor stage, tumor grade as variable, and did not found the independently prognostic value of *PIK3CA* mutation in these factors.

## Discussion

In our study, we investigated the expression of proteins in PI3k pathway, molecular alteration in *PIK3CA* and its impact on survival in patients with NSCLC. To the best of our knowledge, this is the largest cohort of multiple analysis *PIK3CA* gene alteration and the activity of PI3K pathway and we found a negative prognostic impact of single *PIK3CA* mutation in NSCLC. Our study showed frequent overlap of *PIK3CA* and *EGFR/KRAS* mutations and demonstrated a poor prognostic value of *PIK3CA* mutation on *EGFR/KRAS* wildtype patients.

The frequency of *PIK3CA* mutation, as determined by direct sequencing was 3.9% in lung squamous cell carcinoma and 2.7% in adenocarcinoma, which is comparable to the value of 2.9% and 2.5% in a previous report which examined a small number of Japanese patients [21]. Interestingly, in contrast to *PIK3CA*-*KRAS* co-mutation, which is more prevalent in Western countries [28], half of the *PIK3CA* mutant patients in our study had simultaneous *EGFR* mutations. This may be attributed to the higher prevalence *EGFR* mutations in lung cancer patients from East than *KRAS* mutations [17], [29]. Moreover, although PI3K could be activated by receptor kinases and Ras, which in turn activated p-Akt, the PI3K/Akt pathway and *EGFR* signaling pathways interacted closely, PI3K signaling might have additional activators and downstream targets [11], [30]. Our findings on coexistence of *PIK3CA* and other gene mutations within *EGFR* signaling pathways are consistent with these observations.

Consistent with previous studies, high expression of PI3K p110α, p-Akt, mTOR and loss of PTEN was found in 79.4%, 52.9%, 73.5%, 23.5% of *PIK3CA* mutant group and 38.9%, 31.5%, 41.7%, 27.8% of *PIK3CA* wildtype group [20], [26], [27], [31], [32]. We observed that the presence *PIK3CA* mutation was associated with high expression of PI3K p110α, p-Akt, mTOR in NSCLC, similar to the results from ovarian clear cell carcinoma [31]. Nonetheless, one recent research on colorectal cancer reported *PIK3CA* mutation was not in accordance with the expression of PI3K p110α protein, indicating that *PIK3CA* mutations might not be the unique cause leading to high expression of PI3K p110α and might play diverse roles on the activity of PI3K pathway in distinct types of carcinomas [26]. We also demonstrated that PI3K p110α expression was positively correlated with the expression of p-Akt and mTOR, while p-Akt expression was also positively correlated with mTOR expression both in *PIK3CA* mutant group and *PIK3CA* wild type group. These results can be easily understood because that *PIK3CA* activates p-Akt, and positively regulates mTOR [27], [31]. A study by Marsit et al. suggested that regulation of PTEN is not always at the genetic level but also may occur at the transcriptional or translational level, which might also explain the limited correlation between PTEN loss and *PIK3CA* mutation observed [12]. Subsequent association analysis demonstrated that *PIK3CA* mutant tumors with high expression of PI3K p110α were more likely to be stage II to IV disease compared to tumors without high PI3K p110α expression. The expression of PI3K p110α was also found to be correlated with primary and metastatic lesions, suggesting that PI3K p110α might be involved in tumor progression and metastases [25], [26]. The exact molecular mechanism still warrants further study.

To extend our understanding of the mechanism beneath the activation of PI3K pathway, we investigated the correlation between *PIK3CA* amplification and clinicopathological variables in the same serial of NSCLC patients. Similar to the previous studies, we found that *PIK3CA* amplification was significantly associated with smoking history and histologic type, which was more prevalent in smokers compared to never smokers, and in squamous cell carcinomas compared to adenocarcinomas in *PIK3CA* wildtype group [33], [34]. Of note, in the present study, *PIK3CA* amplification was not associated with expression of proteins in PI3K pathway, which was similar to previous studies [11], [35]. These observations suggested *PIK3CA* amplification may not lead to activation of PI3K pathway in any types of lung cancer, insofar as it might be only one of the many downstream targets of PI3-kinase [34].

So far, the impact of *PIK3CA* mutations on survival is still contradictory. Some investigators reported better prognosis in certain cancers such as breast cancer with *PIK3CA* mutations, whereas others suggested that *PIK3CA* mutations indicated a worse prognosis in colorectal cancer, endometrial cancer and NSCLC [36], [37]. However, these results might not be able to successfully assess the true prognostic impact of *PIK3CA* mutation, because survival analysis according to *PIK3CA* mutation status was performed without consideration of the coexistence of *PIK3CA* and other oncogene mutation. In our study, patients with single *PIK3CA* mutation exhibited a shorter OS and RFS than those with *PIK3CA*-*EGFR/KRAS* co-mutation or those in *PIK3CA* wild type group. One plausible explanation was that *PIK3CA* mutation alone might be a prognostic marker for worse survival, however, patients with other oncogene mutations might be more likely to benefit from tyrosine kinase inhibitor therapy.

Furthermore, although *EGFR* mutation was found to be associated with good recurrence-free survival and *KRAS* mutation was shown as a poor prognosis factor, few predictive biomarkers were reported on *EGFR/KRAS* wildtype subgroup [38], [39]. Our study demonstrated a poor survival of *PIK3CA* mutation on patients without *EGFR* or *KRAS* mutations, confirming the prognostic role of *PIK3CA* mutation in *EGFR/KRAS* wildtype subgroup. In addition, as patients in E20 group had a longer OS and RFS than those in E9 group, studies in breast cancer had found clinical significance between patients with mutations in helical (exon 9) and kinase (exon 20) domain with inferior overall survival in those with mutation in the helical domain [40].These observations are also supported by findings in soft tissue sarcoma in which downstream activation level of PI3K is higher in tumors with helical domain mutations than those with kinase domain mutation [41].

Recently, the presence of co-occurrence of *EGFR* and other gene lesions was reported to reduce the sensitivity of *EGFR*-TKI [42], and the addition of a constitutively active PI3K mutant has been shown to confer gefitinib resistance in vitro [43]. Furthermore, it was also demonstrated that *PIK3CA* mutations in exon 20 were associated with resistance to EGFR-targeting monoclonal antibodies [44]. Thus the detection of *PIK3CA* mutation status and its coexistence with other gene mutation would be helpful to predict response to target therapy.

The strength of this study is the comprehensive analysis of *PIK3CA* gene alteration and the prognostic value of distinct *PIK3CA* mutation status in NSCLC. However, we fail to find the independently prognostic role of *PIK3CA* mutation after Cox regression. The sample size of in this series is relative small to draw definitive conclusions. Long term follow-up and larger studies or combinations of experience of multiple institutions will be helpful in clarifying whether this is a true association.

In conclusion, we demonstrated a high frequency of *PIK3CA* and *EGFR/KRAS* co-mutation in NSCLC and a negative prognostic value of *PIK3CA* mutation in *EGFR/KRAS* wildtype subgroup, indicating that different *PIK3CA* mutation status might contribute to distinct therapeutic targets in NSCLC. Further study exploring the *PIK3CA* alteration in a larger scale of population is warranted.

## Supporting Information

File S1 **Supporting information.** **Figure S1** Representative *PIK3CA* mutations by direct sequencing are shown for exon 9(A) and exon 20(B). **Figure S2** Representative high expression of PI3K P110α, p-Akt, m TOR and low expression of PTEN protein in lung SCC; original amplification, ×200 (A); Representative high expression of PI3K, p-Akt, m TOR and PTEN protein in lung AD; original amplification, ×200 (B); Representative cell nuclei having normal(C), gain (D), and amplified (E) *PIK3CA* signals (red) and two centromeric signals (green). **Figure S3** Overall survival curves for patients with or without adjuvant chemotherapy (A); Recurrence-free survival curves for patients with or without adjuvant chemotherapy (B). **Figure S4** Overall survival curves for patients with or without *PIK3CA* amplification (A); Recurrence-free survival curves for patients with or without *PIK3CA* amplification (B). **Table S1** Comparison of *PIK3CA* helical(exon9) and kinase(exon20) domain mutation. **Table S2** *ALK* rearrangement in 1117 NSCLC patients. **Table S3** Correlations between *PIK3CA* mutations and other gene alterations in lung adenocarcinoma. **Table S4** Correlations between *PIK3CA* mutation*s* and other gene alterations in lung squamous cell carcinoma. **Table S5** Comparison of patients with single *PIK3CA* mutation to those with *PIK3CA* and other oncogene mutation. **Table S6** Histopathological subtype in 785 *PIK3CA* wildtype and 22 *PIK3CA* mutant patients with lung adenocarcinoma. **Table S7** Comparison of histopathological subtype between lung adenocarcinoma patients only with *PIK3CA* mutation and those co-exited with *EGFR/KRAS* mutation. **Table S8** Associations of PI3K p110 α, p-Akt, mTOR, PTEN expression and *PIK3CA* amplification with clinicopathologic characteristics of 34 patients in *PIK3CA* mutant group. **Table S9** Associations of PI3K p110 α, p-Akt, mTOR, PTEN expression and *PIK3CA* amplification with clinicopathologic characteristics of 108 patients in *PIK3CA* wild-type group. **Table S10** Associations of PI3K p110α, p-Akt, mTOR, PTEN expression and *PIK3CA* amplification with clinicopathologic characteristics.
(PDF)
