# Supplementary material for: PIK3CA Mutations Frequently Coexist with EGFR/KRAS Mutations in Non-Small Cell Lung Cancer and Suggest Poor Prognosis in EGFR/KRAS Wildtype Subgroup
Source: PLoS One. 2014 Feb 12;9(2):e88291. doi: 10.1371/journal.pone.0088291 (PMC3922761; doi:10.1371/journal.pone.0088291)
Supplement: File S1 — Supporting information. Figure S1 Representative PIK3CA mutations by direct sequencing are shown for exon 9(A) and exon 20(B). Figure S2 Representative high expression of PI3K P110α, p-Akt, m TOR and low expression of PTEN protein in lung SCC; original amplification, ×200 (A); Representative high expression of PI3K, p-Akt, m TOR and PTEN protein in lung AD; original amplification, ×200 (B); Representative cell nuclei having normal(C), gain (D), and amplified (E) PIK3CA signals (red) and two centromeric signals (green). Figure S3 Overall survival curves for patients with or without adjuvant chemotherapy (A); Recurrence-free survival curves for patients with or without adjuvant chemotherapy (B). Figure S4 Overall survival curves for patients with or without PIK3CA amplification (A); Recurrence-free survival curves for patients with or without PIK3CA amplification (B). Table S1 Comparison of PIK3CA helical(exon9) and kinase(exon20) domain mutation. Table S2 ALK rearrangement in 1117 NSCLC patients. Table S3 Correlations between PIK3CA mutations and other gene alterations in lung adenocarcinoma. Table S4 Correlations between PIK3CA mutations and other gene alterations in lung squamous cell carcinoma. Table S5 Comparison of patients with single PIK3CA mutation to those with PIK3CA and other oncogene mutation. Table S6 Histopathological subtype in 785 PIK3CA wildtype and 22 PIK3CA mutant patients with lung adenocarcinoma. Table S7 Comparison of histopathological subtype between lung adenocarcinoma patients only with PIK3CA mutation and those co-exited with EGFR/KRAS mutation. Table S8 Associations of PI3K p110 α, p-Akt, mTOR, PTEN expression and PIK3CA amplification with clinicopathologic characteristics of 34 patients in PIK3CA mutant group. Table S9 Associations of PI3K p110 α, p-Akt, mTOR, PTEN expression and PIK3CA amplification with clinicopathologic characteristics of 108 patients in PIK3CA wild-type group. Table S10 Associations of PI3K p110α, p-Akt, mTOR, PTEN exp [file pone.0088291.s001.pdf]

Figure S1

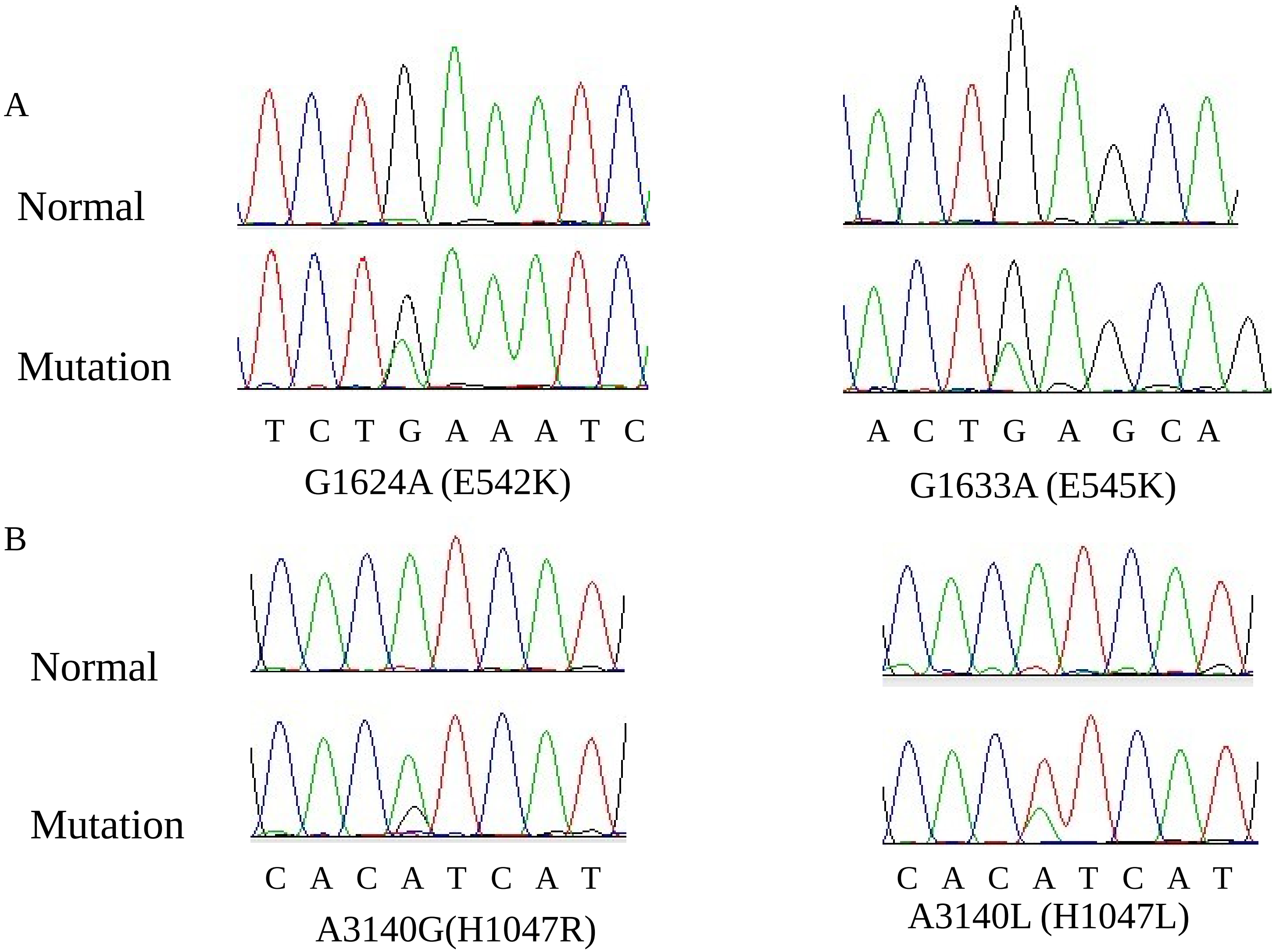
A
Normal
Mutation
T C T G A A A T C
G1624A (E542K)
A C T G A G C A
G1633A (E545K)
B
Normal
Mutation
C A C A T C A T
A3140G(H1047R)
C A C A T C A T
A3140L (H1047L)

Figure S2

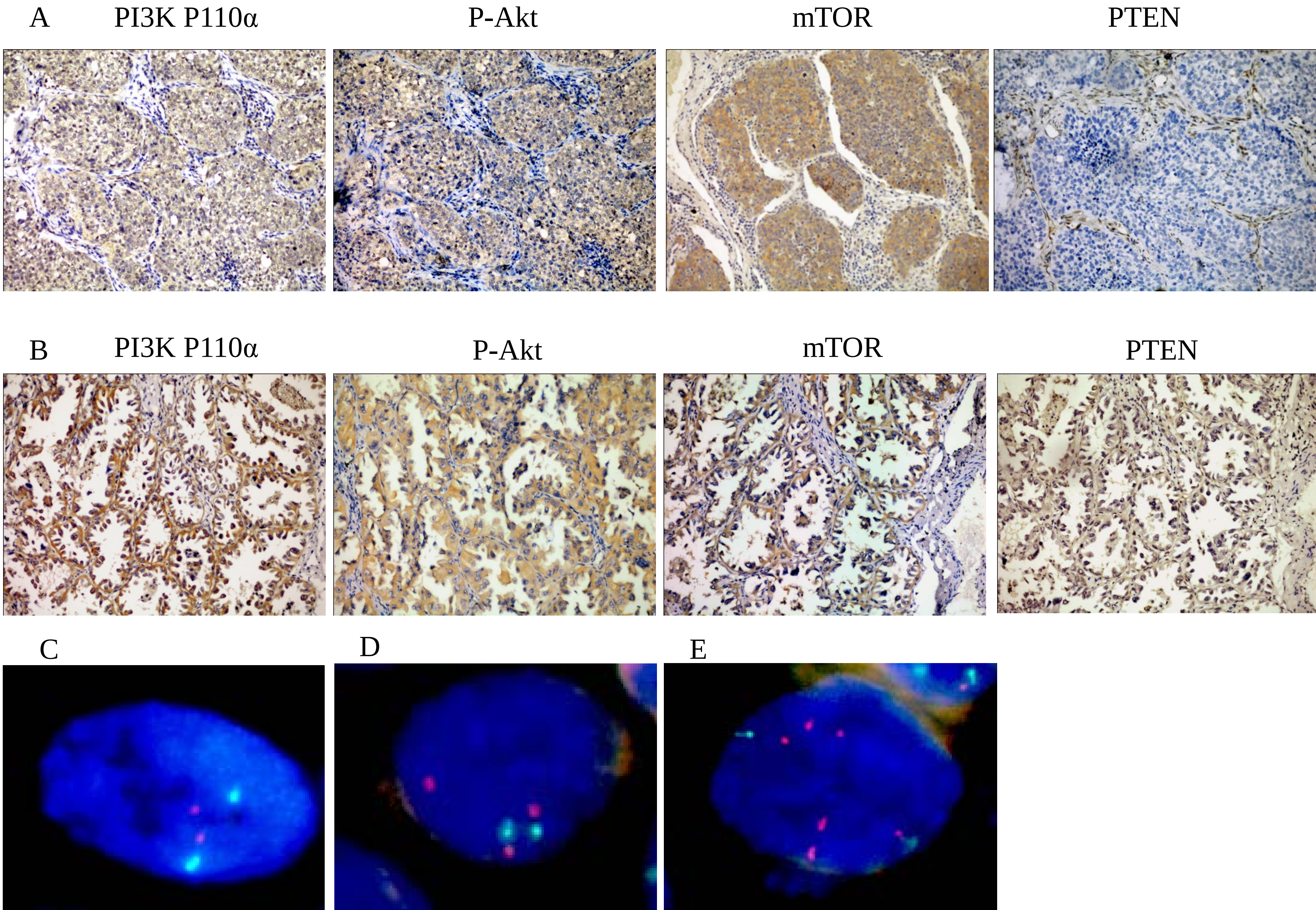

Figure S3

A

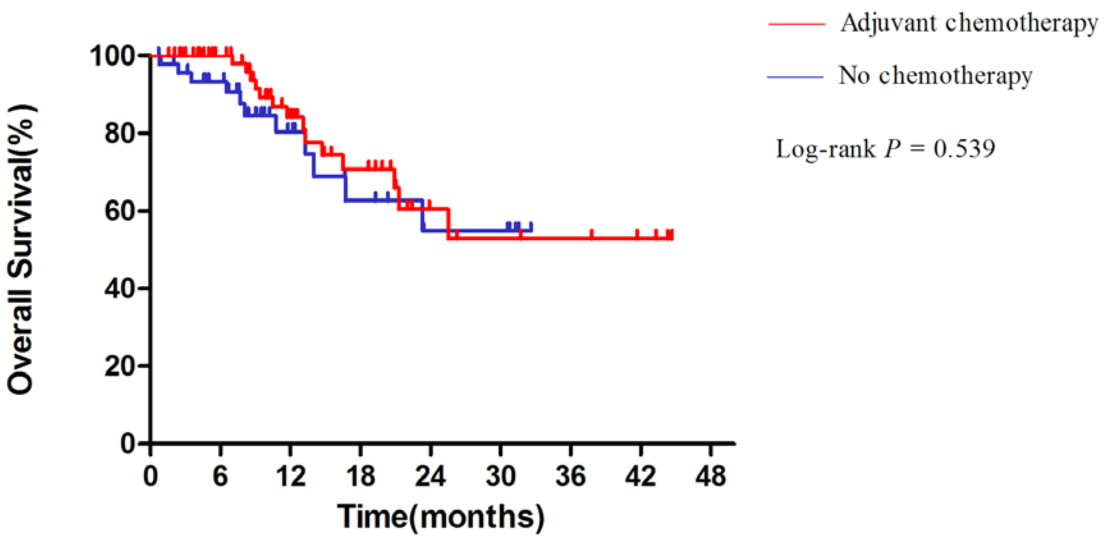
Adjuvant chemotherapy
No chemotherapy
Log-rank P = 0.539
Overall Survival(%)
100
80
60
40
20
0
0
6
12
18
24
30
36
42
48
Time(months)

B

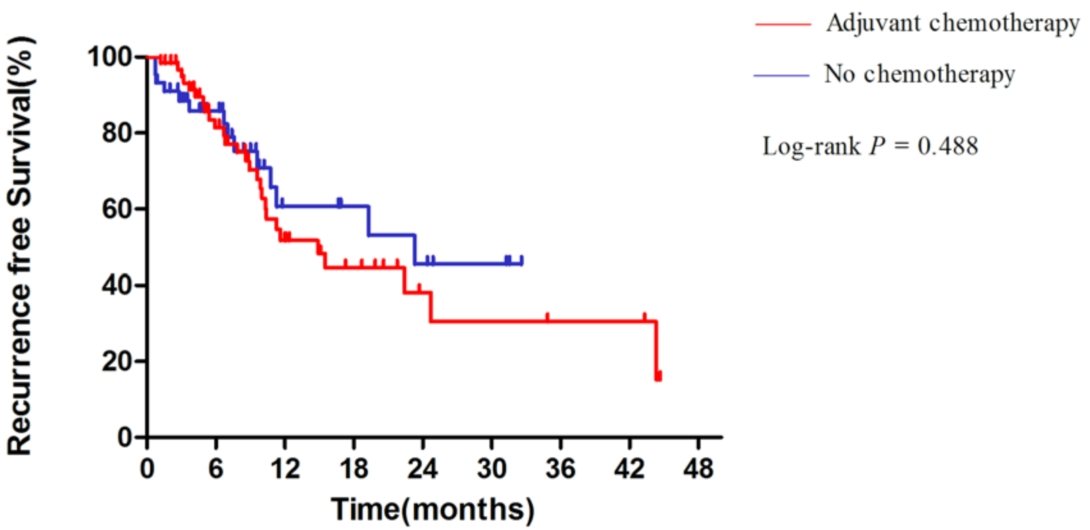
Adjuvant chemotherapy
No chemotherapy
Log-rank P = 0.488
Recurrence free Survival(%)
100
80
60
40
20
0
0
6
12
18
24
30
36
42
48
Time(months)

Figure S4

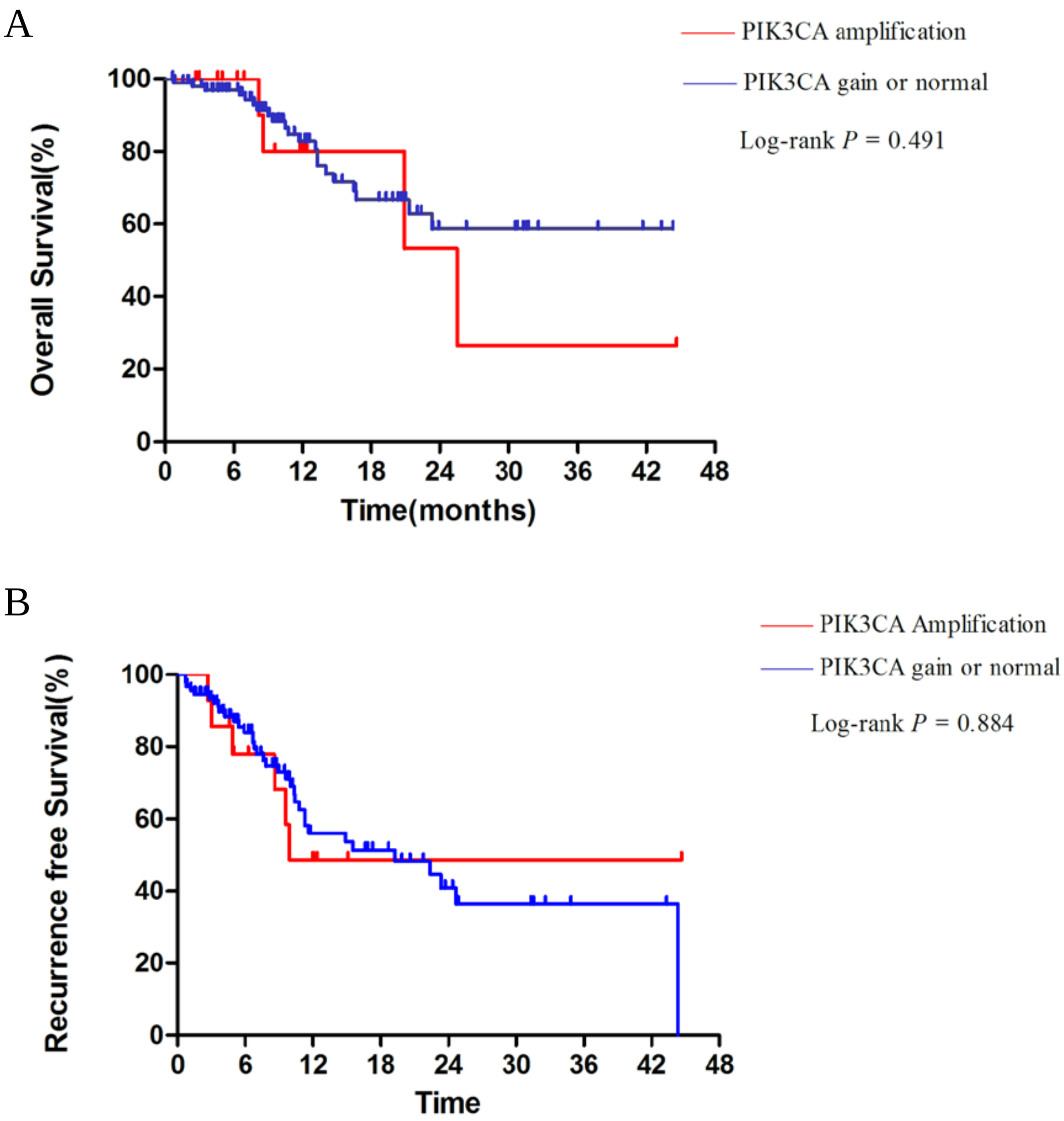

A
PIK3CA amplification
PIK3CA gain or normal
Log-rank P = 0.491
Overall Survival(%)
100
80
60
40
20
0
0
6
12
18
24
30
36
42
48
Time(months)
B
PIK3CA Amplification
PIK3CA gain or normal
Log-rank P = 0.884
Recurrence free Survival(%)
100
80
60
40
20
0
0
6
12
18
24
30
36
42
48
Time

Table S1 Comparison of *PIK3CA* helical(exon9) and kinase(exon20) domain mutation

Characteristics	Helical N (%)	Kinase N (%)	*p*-value
Stage			
Ⅰ	6(28.6)	7(53.8)	
Ⅱ~Ⅳ	15(71.4)	6(46.2)	0.141
Lymph node metastasis			
N0	8(38.1)	7(53.8)	0.369
N+	13(61.9)	6(46.2)	
Smoking history			
Never	9(42.9)	7(53.8)	
Former/current	12(57.1)	6(46.2)	0.533
Differentiation			
Well	2(9.5)	2(15.4)	0.606
Moderately or poorly	19(90.5)	11(84.6)	
Pathological types			
AD	13(61.9)	9(69.2)	0.727
SCC	8(38.1)	4(30.8)	
Age			
≤60	11(52.4)	9(69.2)	0.477
>60	10(47.6)	4(30.8)	
Gender			
Male	15(71.4)	6(46.2)	0.141
Female	6(28.6)	7(53.8)	
Co-mutation			
Yes	13(61.9)	8(61.5)	
No	8(38.1)	5(38.5)	0.983

N+: lymph node metastasis positive; AD: adenocarcinoma; SCC:squamous cell carcinoma

Table S2 *ALK* rearrangement in 1117 NSCLC patients

Patients ID	Pathology	PCR	FISH
245	AD	E13;A20	+
324	AD	E20;A20	+
347	AD	E20;A20	+
399	AD	E6a/b;A20	+
460	AD	E13;A20	+
490	AD	E6a/b;A20	+
578	AD	E13;A20	+
584	AD	E17;A20	+
591	AD	E6a/b;A20	+
636	AD	E13;A20	+
667	AD	E13;A20	+
732	AD	E20;A20	+
737	AD	E17 ins 65;A20	+
781	AD	E6b;A20	+
782	AD	E6a/b;A20	+
788	AD	E6a/b;A20	+
834	AD	E13;A20	+
877	AD	E20;A20	+
964	AD	E20;A20	+
966	AD	E20;A20	+
974	AD	E13;A20	+
981	AD	E17b ins 39;A20	+
988	AD	E10a/b, E13;A20	+
1003	AD	E13;A20	+
1004	AD	E13;A20	+
1228	AD	E13;A20	+
1335	AD	E13;A20	+
1336	AD	E13;A20	+
1451	AD	E13;A20	+
1459	AD	E13:A20	+
1494	AD	(E6a/b:ALK intron 18bp:A20	+
1514	AD	E6a/b;A20	+
B56	AD	E6a/b;A20	+

NOTE: +, positive; AD, adenocarcinoma;

Table S3 Correlations between *PIK3CA* mutations and other gene alterations in lung adenocarcinoma.

Gene alteration		*PIK3CA* mutant N (%)	*PIK3CA* wildtype N (%)	*p*-Value
EGFR	mutant	16(72.7)	506(64.5)	
	wildtype	6(27.3)	279(35.5)	0.424
KRAS	mutant	3(13.6)	56(7.1)	
	wildtype	19(86.4)	729(92.9)	0.213
HER2	mutant	0(0.0)	19(2.4)	
	wildtype	22(100.0)	766(97.6)	1.000
BRAF	mutant	0(0.0)	10(1.3)	
	wildtype	22(100.0)	775(98.7)	1.000
AKT1	mutant	0(0.0)	1(0.1)	
	wildtype	22(100.0)	784(99.9)	1.000
ALK	rearrangement	0(0.0)	31(3.9)	
	wildtype	22(100.0)	754(96.1)	1.000

Table S4 Correlations between *PIK3CA* mutations and other gene alterations in lung squamous cell carcinoma

Gene alteration		*PIK3CA* mutant N (%)	*PIK3CA* wildtype N (%)	*p*-Value
EGFR	mutant	1(8.3)	13(4.4)	
	wildtype	11(91.7)	285(95.6)	0.432
KRAS	mutant	1(8.3)	7(2.3)	
	wildtype	11(91.7)	291(97.7)	0.274
HER2	mutant	0(0.0)	1(0.3)	
	wildtype	12(100.0)	297(99.7)	1.000
BRAF	mutant	0(0.0)	1(0.3)	
	wildtype	12(100.0)	297(97.7)	1.000
AKT1	mutant	0(0.0)	1(0.3)	
	wildtype	12(100.0)	297(97.7)	1.000
ALK	rearrangement	0(0.0)	2(0.7)	
	wildtype	12(100.0)	296(99.3)	1.000

Table S5 Comparison of patients with single *PIK3CA* mutation to those with *PIK3CA* and other oncogene mutation

Characteristics	Coexistence of *PIK3CA* and *EGFR/KRAS* mutations N(%)	Patients with *PIK3CA* mutation only N(%)	*p*-value
Stage			
Ⅰ	8(38.1)	4(30.8))	0.727
Ⅱ~Ⅳ	13(61.9)	9(69.2)	
Smoking history			
Never smoker	13(61.9)	3(23.1)	0.039
Current or former smoker	8(38.1)	10(76.9)	
Pathological types			
AD	19(90.5)	3(23.1)	**<0.0001**
SCC	2(9.5)	10(76.9)	
Age			
≤60	15(71.4)	6(46.2)	0.168
>60	6(28.6)	7(53.8)	
Gender			
Male	10(47.6)	11(84.6)	0.067
Female	11(52.4)	2(15.4)	
Domain			
Helical domain	13(61.9)	8(61.5)	1.000
Kinase domain	8(38.1)	5(38.5)	

AD: adenocarcinoma; SCC: squamous cell carcinoma

Table S6 Histopathological subtype in 785 *PIK3CA* wildtype and 22 *PIK3CA* mutant patients with lung adenocarcinoma

Histologic subtype	*PIK3CA* mutations patients N (%)	*PIK3CA* Wild type patients N (%)	*p*-Value
AIS	1(4.5)	5(0.6)	
MIA	0(0.0)	13(1.5)	
Lepidic predominant	2(9.1)	53(6.8)	
Acinar predominant	11(50)	412(52.5)	
Papillary predominant	1(4.5)	121(15.4)	
Micropapillary predominant	0(0.0)	16(2.0)	
Solid predominant	6(27.3)	137(17.5)	
IMA	1(4.5)	25(3.2)	
Enteric predominant	0(0.0)	3(0.4)	0.082

AIS: adenocarcinoma in situ; MIA: minimally invasive adenocarcinoma;
IMA:invasive mucinous adenocarcinoma

Table S7 Comparison of histopathological subtype between lung adenocarcinoma patients only with *PIK3CA* mutation and those co-exited with *EGFR/KRAS* mutation

Histologic subtype	Patients with *PIK3CA* and *EGFR/KRAS* mutations N(%)	Patients with *PIK3CA* mutation only N(%)	*p*-Value
AIS	0(0.0)	1(33.3)	
Lepidic predominant	2(10.5)	0(0.0)	
Acinar predominant	10(52.6)	1(33.3)	
Papillary predominant	1(5.3)	0(0.0)	
Solid predominant	5(26.3)	1(33.3)	
IMA	1(5.3)	0(0.0)	0.121

AIS: adenocarcinoma in situ; IMA:invasive mucinous adenocarcinoma

Table S8 Associations of PI3K p110 α, p-Akt, mTOR, PTEN expression and *PIK3CA* amplification with clinicopathologic characteristics of 34 patients in *PIK3CA* mutant group

Characteristics	PI3K p110 α			PTEN loss			p-Akt			m TOR		
	+(%)	-(%)	*p* value	+(%)	-(%)	*p* value	+(%)	-(%)	*p* value	+(%)	-(%)	*p* value
Stage												
Ⅰ	8(61.5)	5(38.5)	**0.043**	2(15.4)	11(84.6)	0.444	8(61.5)	5(38.5)	0.429	8(61.5)	5(38.5)	0.254
Ⅱ~Ⅳ	19(90.5)	2(9.5)		6(28.6)	15(71.4)		10(47.6)	11(52.4)		17(81.0)	4((19.0)	
Lymph node metastasis												
N0	11(73.3)	4(26.7)	0.672	3(20.0)	12(80.0)	1.000	10(66.7)	5(33.3)	0.154	11(73.3)	4((26.7)	1.000
N+	16(84.2)	3(15.8)		5(26.3)	14(73.7)		8(42.1)	11((57.9)		14(73.7)	5(26.3)	
Smoking history												
Never smoker	11(68.8)	5(31.3)	0.214	2(12.5)	14(87.5)	0.233	10(62.5)	6(37.5)	0.327	12(75.0)	4(25.0)	1.000
C/F smoker	16(88.9)	2(11.1)		6(33.3)	12(66.7)		8(44.4)	10(55.6)		13(72.2)	5((27.8)	
Differentiation												
Well	3(75.0)	1(25.0)	1.000	1(25.0)	3(75.0)	1.000	1(25.0)	3(75.0)	0.323	3(75.0)	1(25.0)	0.610
Moderately or poorly	24(80.0)	6(20.0)		7(23.3)	23(76.7)		17(56.7)	13(43.2)		22(73.3)	8(26.7)	
Pathological types												
AD	17(77.3)	5(22.7)	1.000	4(18.2)	18(81.8)	0.410	12(54.5)	10(15.5)	0.800	15(68.2)	7(31.8)	0.439
SCC	10(83.3)	2(16.7)		4(33.3)	8(66.7)		6(50.0)	6(50.0)		10(83.3)	2(16.7)	
Age												
≤60	15(71.4)	6(28.6)	0.210	5(23.8)	16(76.2)	0.262	12(57.1)	9(42.9)	0.533	15(71.4)	6(28.6)	1.000
>60	12(92.3)	1(7.7)		6(46.2)	7(53.8)		6(46.2)	7(53.8)		10(76.9)	3(23.1)	
Gender												
Male	18(85.7)	3(14.3)	0.387	6(28.6)	15(71.4)	0.444	11(52.4)	10(47.6)	0.934	16(76.2)	5(23.8)	0.704
Female	9(69.2)	4(30.8)		2(15.4)	11(84.6)		7(53.8)	6(46.2)		9(69.2)	4(30.8)	
PI3K p110α												
+	NA			7(25.9)	20(74.1)	1.000	17(63.0)	10(37.0)	**0.035**	23(85.2)	4(14.8)	**0.007**
-				1(14.3)	6(85.7)		1(14.3)	6(85.7)		2(28.6)	5(71.4)	
PTEN loss												
+	7(87.5)	1(12.5)	1.000	NA			4(50.0)	4(50.0)	1.000	7(87.5)	1(12.5)	0.403
-	20(76.9)	6(23.1)					14(53.8)	12(46.2)		18(69.2)	8(30.8)	
p-Akt												
+	17(94.4)	1(5.6)	**0.035**	4(22.2)	14(77.8)	1.000	NA			18(100.0)	0(0.0)	**<0.0001**
-	10(62.5)	6(37.5)		4(25.0)	12(75.0)					7(43.8)	9(56.3)	
m TOR												
+	23(92.0)	2(8.0)	**0.007**	7(28.0)	18(72.0)	0.403	18(72.0)	7(28.0)	**<0.0001**	NA		
-	4(44.4)	5(55.6)		1(11.1)	8(88.9)		0(0.0)	9(100.0)				

N+: lymph node metastasis positive; C/F: current/former; AD: adenocarcinoma;SCC;squamous cell carcinoma; NA: Not applicable

Table S9 Associations of PI3K p110 α, p-Akt, mTOR, PTEN expression and *PIK3CA* amplification with clinicopathologic characteristics of 108 patients in *PIK3CA* wild-type group

Characteristics	PI3K p110 α			PTEN loss			p-Akt			m TOR		
	+(%)	-(%)	*p* value	+(%)	-(%)	*p* value	+(%)	-(%)	*p* value	+(%)	-(%)	*p* value
Stage												
Ⅰ	8(29.6)	19(70.4)		5(18.5)	22((81.5)		8(29.6)	19(70.4)		13(48.1)	14(51.9)	
Ⅱ~Ⅳ	33(40.7)	48(59.3)	0.303	23(28.4)	58(71.6)	0.310	25(30.9)	56(69.1)	0.904	32(39.5)	49(60.5)	0.430
Lymph node metastasis												
N0	23(43.4)	30(56.6)	0.253	18(34.0)	35(66.0)	0.061	17(32.1)	36(67.9)	0.736	25(47.2)	28(52.8)	0.255
N+	18(32.7)	37(67.2)		10(18.2)	45(81.8)		16(29.1)	39(70.9)		20(36.4)	35(63.6)	
Smoking history												
Never smoker	12(40.0)	18(60.0)	0.787	4(13.3)	26(86.7)	0.064	8(26.7)	22(73.3)	0.586	13(43.3)	17(56.7)	0.528
C/F smoker	29(37.2)	49(62.8)		24(30.8)	54(69.2)		25(32.1)	53(67.9)		32(41.0)	46(59.0)	
Differentiation												
Well	1(20.0)	4(80.0)	0.648	2(40.0)	3(60.0)	0.603	1(20.0)	4(80.0)	1.000	3(60.0)	2(40.0)	0.647
Moderately or poorly	40(38.8)	63(61.2)		26(25.2)	77(74.8)		32(31.1)	71(68.9)		42(40.8)	61(59.2)	
Pathological types												
AD	22(39.3)	34(60.7)	0.769	13(23.2)	43(76.8)	0.505	15(26.8)	41(73.2)	0.377	22(39.3)	34(60.7)	0.602
SCC	19(36.5)	33(63.5)		15(28.8)	37(71.2)		18(34.6)	34(65.4)		23(44.2)	29(55.8)	
Age												
≤60	21(42.9)	28(57.1)	0.340	11(22.4)	38(77.6)	0.452	10(20.4)	39(79.6)	**0.037**	19(38.8)	30(61.2)	0.579
>60	20(33.9)	39(66.1)		17(28.8)	42(71.2)		23(39.0)	36(61.0)		26(44.1)	33(55.9)	
Gender												
Male	34(37.8)	56(62.2)	0.929	24(26.7)	66(73.3)	0.694	30(33.3)	60(66.7)	0.161	36(40.0)	54(60.0)	0.579
Female	7(42.2)	11(61.1)		4(22.2)	14(77.8)		3(16.7)	15(83.3)		9(50.0)	9(50.0)	
PI3K p110 α												
+	NA			7(17.1)	34(82.9)	0.101	23(56.1)	18(43.9)	**<0.0001**	32(78.0)	9(22.0)	**<0.0001**
-				21(31.3)	46(68.7)		10(14.9)	57(85.1)		13(19.4)	54(80.6)	
PTEN loss												
+	7(25.0)	21(75.0)	0.101	NA			7(25.0)	21(75.0)	0.458	8(28.6)	20(71.4)	0.102
-	34(42.5)	46(57.5)					26(32.5)	54(67.5)		37(46.3)	43(53.8)	
p-Akt												
+	23(69.7)	10(30.3)	**<0.0001**	7(21.2)	26(78.8)	0.458	NA			21(63.6)	12(36.4)	**0.002**
-	18(24.0)	57(76.0)		21(28.0)	54(72.0)					24(32.0)	51(68.0)	
m TOR												
+	32(71.1)	13(28.9)	**<0.0001**	8(17.8)	37(82.2)	0.102	21(46.7)	24(53.3)	**0.002**	NA		
-	9(14.3)	54(85.7)		20(31.7)	43(68.3)		12(19.0)	51(81.0)				

N+: lymph node metastasis positive; C/F: current/former; AD: adenocarcinoma; SCC;squamous cell carcinoma; NA: Not applicable

Table S10 Associations of PI3K p110α, p-Akt , mTOR, PTEN expression and *PIK3CA* amplification with clinicopathologic characteristics

Characteristics	*PIK3CA* amplification in *PIK3C*A mutant group			*PIK3CA* amplification in *PIK3CA* wildtype group		
	+(%)	-(%)	*P* value	+(%)	-(%)	*P* value
Stage						
Ⅰ	2(15.4)	11(84.6)	1.000	6(22.2)	21(77.8)	0.783
Ⅱ~Ⅳ	3(14.3)	18(85.7)		16(19.8)	65(80.2)	
Lymph node metastasis						
N0	3(20.0)	12(80.0)	0.634	12(22.6)	41(77.4)	0.565
N+	2(10.5)	17(89.5)		10(18.2)	45(81.8)	
Smoking history						
Never smoker	2(12.5)	14(87.5)	1.000	0(0)	30(100.0)	**0.001**
Current/former smoker	3(16.7)	15(83.3)		22(28.2)	56(71.8)	
Differentiation						
Well	1(25.0)	3(75.0)	0.488	0(0)	5(100.0)	0.581
Moderately or poorly	4(13.3)	26(86.7)		22(21.4)	81(78.6)	
Pathological types						
AD	1(4.5)	21(95.5)	**0.042**	3(5.4)	53(94.6)	**<0.0001**
SCC	4(33.3)	8(66.7)		19(36.5)	33(63.5)	
Age						
≤60	2(9.5)	19(90.5)	0.348	8(16.3)	41(83.7)	0.342
>60	3(23.1)	10(76.9)		14(23.7)	45(76.3)	
Gender						
Male	4(19.0)	17(81.0)	0.627	22(24.4)	68(75.6)	**0.021**
Female	1(7.7)	12(92.3)		0(0)	18(100.0)	
PI3K p110α						
+	5(18.5)	22(81.5)	0.559	7(17.1)	34(82.9)	0.506
-	0(0)	7(100.0)		15(22.4)	52(77.6)	
PTEN loss						
+	2(25.0)	6(75.0)	0.570	7(25.0)	21(75.0)	0.480
-	3(11.5)	23(88.5)		15(18.8)	65(81.3)	
p-Akt						
+	4(22.2)	14(77.8)	0.340	6(18.2)	27(81.8)	0.708
-	1(6.3)	15((93.8)		16(21.3)	59(78.7)	
m TOR						
+	5(20.0)	20(80.0)	0.293	10(22.2)	35(77.8)	0.686
-	0(0)	9(100.0)		12(19.0)	51(81.0)	

N+: lymph node metastasis positive AD: adenocarcinoma;SCC;squamous cell carcinoma

Supporting information legend:

Figure S1 Representative *PIK3CA* mutations by direct sequencing are shown for exon 9(A) and exon 20(B).

Figure S2 Representative high expression of PI3K P110α, p-Akt, m TOR and low expression of PTEN protein in lung SCC; original amplification, ×200 (A); Representative high expression of PI3K, p-Akt, m TOR and PTEN protein in lung AD; original amplification, ×200 (B); Representative cell nuclei having normal(C), gain (D), and amplified (E) *PIK3CA* signals (red) and two centromeric signals (green).

Figure S3 Overall survival curves for patients with or without adjuvant chemotherapy (A); Recurrence-free survival curves for patients with or without adjuvant chemotherapy (B).

Figure S4 Overall survival curves for patients with or without *PIK3CA* amplification (A); Recurrence-free survival curves for patients with or without *PIK3CA* amplification (B).

Table S1 Comparison of *PIK3CA* helical(exon9) and kinase(exon20) domain mutation.

Table S2 *ALK* rearrangement in 1117 NSCLC patients

Table S3 Correlations between *PIK3CA* mutations and other gene alterations in lung adenocarcinoma.

Table S4 Correlations between *PIK3CA* mutation*s* and other gene alterations in lung squamous cell carcinoma

Table S5 Comparison of patients with single *PIK3CA* mutation to those with *PIK3CA* and other oncogene mutation

Table S6 Histopathological subtype in 785 *PIK3CA* wildtype and 22 *PIK3CA* mutant patients with lung adenocarcinoma

Table S7 Comparison of histopathological subtype between lung adenocarcinoma patients only with *PIK3CA* mutation and those co-exited with *EGFR/KRAS* mutation

Table S8 Associations of PI3K p110 α, p-Akt, mTOR, PTEN expression and *PIK3CA* amplification with clinicopathologic characteristics of 34 patients in *PIK3CA* mutant group

Table S9 Associations of PI3K p110 α, p-Akt, mTOR, PTEN expression and *PIK3CA* amplification with clinicopathologic characteristics of 108 patients in *PIK3CA* wild-type group

Table S10 Associations of PI3K p110α, p-Akt , mTOR, PTEN expression and *PIK3CA*

amplification with clinicopathologic characteristics
